# Salvaged, Staged, and Safer Management of Aortoesophageal Fistula and Mediastinitis After Removing a Pork Bone: A Case Report

**DOI:** 10.3389/fsurg.2022.916006

**Published:** 2022-06-08

**Authors:** Yan Ge, Ayinuer Tuerdi, Xinming Yang, Jingqun Tang, Quanming Li

**Affiliations:** ^1^ Department of Rheumatology and Immunology, The Second Xiangya Hospital of Central South University, Changsha, China; ^2^ Department of Otolaryngology-Head and Neck Surgery, The Second Xiangya Hospital of Central South University, Changsha, China; ^3^ Department of Thoracic Surgery, The Second Xiangya Hospital of Central South University, Changsha, China; ^4^ Department of Vascular Surgery, The Second Xiangya Hospital of Central South University, Changsha, China

**Keywords:** esophageal foreign body, mediastinitis, thoracic endovascular aortic repair, video-assisted thoracoscopic surgery, salvaged staged strategy, aortoesophageal fistula

## Abstract

Aortoesophageal fistula (AEF) caused by esophageal foreign body (EFB) ingestion is a life-threatening condition with a very low survival rate. However, the optimal management strategy remains undetermined. Here, we describe our successful management of a patient with AEF and mediastinitis. A 36-year-old man developed persistent chest and back pain and vomited fresh blood three days after removal of a pork bone in the esophagus under endoscopy in a local hospital. Computed tomography (CT) confirmed bilateral pulmonary infections, mediastinitis, and fistula of the aortic arch. After a multidiscipline discussion, a comprehensive staged strategy was made including salvaged thoracic endovascular aortic repair (TEVAR) to control fatal bleeding, adequate mediastinal debridement, drainage with cervical incision, and video-assisted thoracoscopic surgery, as well as jejunostomy to prevent nasal or gastrostomy reflux from aggravating the mediastinal infection. Furthermore, systematic personalized nutrition support and antibiotics were provided. The patient recovered well and has survived for 50 months until now. Careful assessment should be made with CT to ascertain the risk of AEF before and after the removal of EFB. A salvaged staged strategy of TEVAR with adequate mediastinal debridement and drainage in a less invasive approach may be a safer alternative for AEF patients with infections caused by EFB.

## Introduction

Accidental ingestion of foreign bodies is a common condition that cannot be resolved in some cases. Since the thoracic esophagus is adjacent to the aorta, the foreign body here is more dangerous, causing serious complications such as esophageal perforation, mediastinitis, aortoesophageal fistula (AEF), or mycotic aortic aneurysms (1). AEF is a life-threatening condition; it has been reported that patients with aortic hemorrhage caused by an esophageal foreign body (EFB) all died after conservative treatment, and the mortality rate of patients undergoing active surgery during hospitalization was as high as 45.4%–55% (2). However, no consensus has been reached on the management of AEF caused by EFB until now. This study presented our successful treatment of a patient with AEF and mediastinitis after the removal of a pork bone with a salvaged, staged, and safer management strategy in a less invasive approach.

## Case Presentation

**Figure 1:**
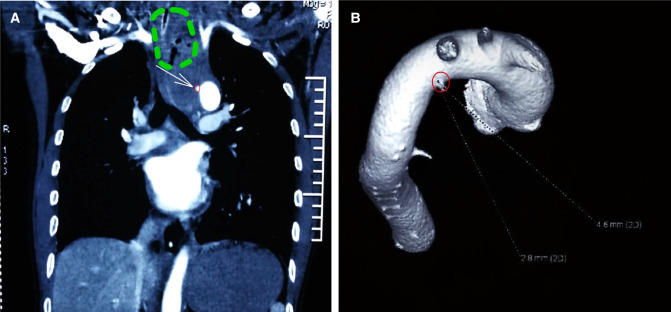
Preoperative examinations. (**A**) Computed tomography angiography (CTA): coronal view of mediastinitis and aortoesophageal fistula. Mediastinitis and gas accumulation with the green circle. The white arrow points to the leakage of contrast from the aorta, indicating a break in the aortic wall, within a small red circle. (**B**) Three-dimensional CT showed a break in the aortic wall (big red circle points to the aortic breakage).

A 36-year-old man underwent the removal of a pork bone in the esophagus using a rigid endoscopy in a local hospital on January 12, 2018. The post-operative course was initially uneventful, and he started eating on the third post-operative day. He then suddenly developed continuous chest and back pain, and vomited a large volume of fresh blood. The computed tomography (CT) images revealed bilateral pulmonary infection and pleural effusion, as well as mediastinitis with gas within the mediastinum (Figure 1A). The findings of three-dimensional CT and aortic computed tomography angiography (CTA) confirmed a fistula of the aortic arch (Figure 1). The patient was transferred to our hospital seven days after the EFB removal.

On admission (day 1), his temperature was 37.6°C, blood pressure was 140/69 mmHg, and heart rate was 94 bpm. The laboratory examination demonstrated leukocytosis (white blood cells, 25.69 × 10^9^/L), neutrophilia (N%, 89.6%), anemia (60 g/L), elevated C-reactive protein (189 mg/L), and procalcitonin (23.38 ng/ml). After an urgent multidisciplinary discussion, a comprehensive staged strategy based on the patient’s condition was made, which was a salvaged thoracic endovascular aortic repair (TEVAR) followed by mediastinal debridement, drainage with cervical incision, and thoracic debridement, drainage with video-assisted thoracoscopic surgery (VATS) combined with an early jejunostomy. Also, adequate nutrition support and antibiotic treatment were provided.

**Figure 2:**
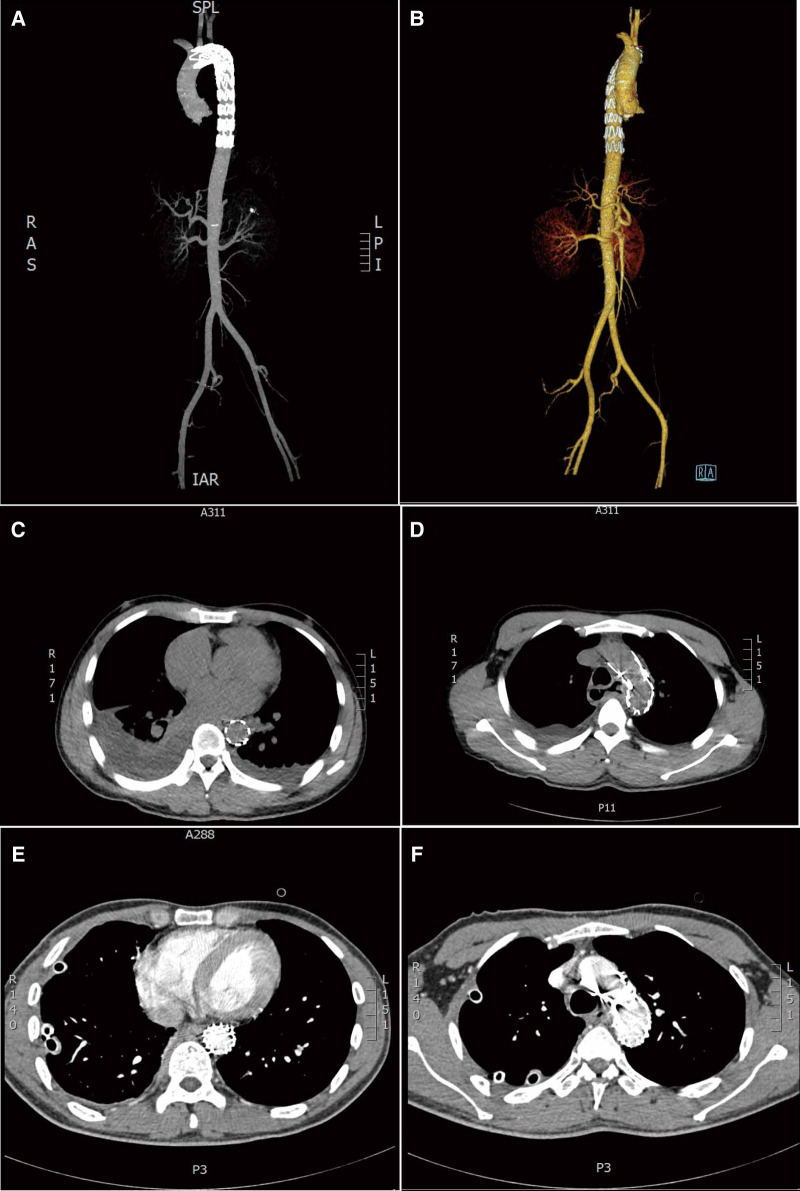
Postoperative images. (**A** and **B**) different views of three-dimensional computed tomography angiography (CTA): complete sealing of the break in the aortic wall after thoracic endovascular aortic repair (TEVAR). (**C**,**D**) Transverse view: complete sealing of the break in the aortic wall, no contrast agent extravasation was observed around the stent after TEVAR; bilateral pleural effusions were seen mainly on the right side. (**E**,**F**) Axial contrast-enhanced CT image after mediastinal debridement and drainage with cervical incision and video-assisted thoracoscopic surgery: the stent and drainage tube were observed clearly, with no fluid on both sides of the chest.

Angiography was performed immediately on day 1, revealing a shadow near the left subclavian artery of the aortic arch. A tapered designed stent graft (180 mm long, proximal-end diameter 28 mm, telecentric diameter 22 mm; Lifetech Scientific, China) was delivered from the right femoral artery and placed at the descending aorta covering the ostium of the left subclavian artery. Intraoperative angiography confirmed successful results. The post-operative CTA showed that the aortic stent was successfully placed and no leakage of contrast agents was found around the stent (Figure 2A–D).

We performed thorough mediastinal debridement and drainage under general anesthesia on day 5. A cervical incision was made along the right sternocleidomastoid anterior rim. At the junction of the common carotid artery and the trachea and sternum, the tissue was blocked by inflammation and the abscess contained a large amount of air and a small amount of pus, which were observed during the surgery. Saline was repeatedly flushed into the abscess, and the drainage tube was placed. The patient then underwent a right VATS exploration for thoracic debridement and drainage. Extensive adhesion was observed in the right thoracic cavity with right pleural effusion and pale yellow and flocculated secretions. In addition, a laparoscopic jejunostomy was performed to prevent nasal or gastrostomy reflux from aggravating infection and enteral nutrition support was provided for the future.

An individualized nutritional plan was developed according to the patient’s condition. In addition to parenteral nutrition, early enteral nutrition was started 24 h after the surgery through a jejunostomy tube and gradually increased until the target amount was reached.

**Figure 3:**
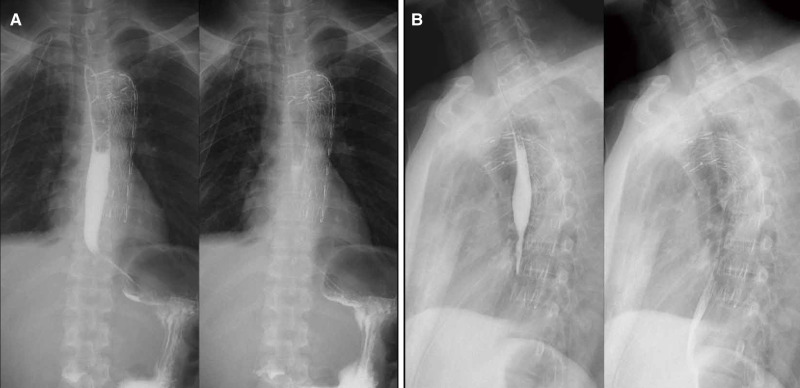
Postoperative upper digestive tract iodine angiography. (**A**,**B**) Esophageal walls were intact, without contrast agent leakage.

On day 11, post-operative contrast-enhanced CT revealed bilateral pleural effusion disappeared, and the drainage tube and the stent were observed clearly (Figure 2E–F). On day 21, an upper digestive tract iodine angiography was performed and confirmed the esophageal fistula had healed (Figure 3), and the patient was switched to tube feeding combined with oral enteral nutrition. The patient was discharged from the hospital on day 24. The patient has remained in good condition after stent implantation until now and has survived for 50 months. Written informed consent was obtained from the patient to publish this case report and any accompanying images.

## Discussion

AEF is a serious, life-threatening complication with a very low survival rate (2). The main causes of AEF are thoracic aneurysms (51%), EFB (19%), esophageal ulcers, and iatrogenic factors such as arterial stent placement (3). Endoscopic treatment can be used to accurately localize and remove foreign bodies and repair esophageal injuries (4). However, it carries the risk of further aortic damage and even fatal bleeding as the foreign body may shift and penetrate the adjacent aortic wall. Thus, it is very important to evaluate the extent of esophageal injuries and predict the risk of AEF for doctors before the removal of EFB. We recommended using multidetector CT to classify esophageal injuries and their risk of AEF, and to guide further treatment combined with the clinical presentation (4). Although there was no obvious bleeding at the time of endoscopy, considering our patient quickly developed the typical symptoms of AEF (chest pain, sentinel hemorrhage, and a symptomless period), we speculate that the patient may have already developed AEF or was at high risk of AEF at that time. A careful examination with CT before and after endoscopic surgery may be helpful to avoid such lethal complications in patients.

Although the first reported survival from AEF caused by a foreign body was recorded by Ctercteko in 1980, the morbidity rate of patients with AEF remains extremely high, and there has been no consensus about the standard treatment (5). Open aortic replacement and primary esophageal repair have previously been regarded as the only effective solutions (6). However, the operative mortality was reported to be as high as 45.4%–55% (7–9). TEVAR is less invasive and enables rapid exclusion of aortic fistula and control of fatal hemorrhage, and has been widely used as the first choice in life-saving procedures in emergencies today (7). However, for patients with AEF, endovascular treatment alone is highly risky because of infection complications, which could result in delayed death (10, 11). In general, it should be considered as a bridge procedure for further treatment or as a temporary alternative until the patient is healthy enough to tolerate open surgery. There have been some cases of the successful use of endograft as a temporary treatment in patients with aortoduodenal and ilioenteric fistulas that allowed improvement of their general condition until definitive treatment by open surgery was possible. However, patients with infected AEFs are still at high risk of mortality from open surgery.

Wei reported that three patients with AEF and mediastinitis caused by EFB survived after endovascular stenting combined with a second-stage VATS exploration for foreign body removal, esophageal repair, and mediastinal and thoracic drainage, while the other three patients, who had received left thoracotomy, esophageal repair, and aortic repair or replacement, died of a hemorrhage within 24 h of the operation (4). Recently, Zhang succeeded in the management of delayed aortoesophageal and tracheoesophageal fistulas secondary to EFB with TEVAR and mediastinal drainage with VATS (12). In addition, a patient with delayed AEF due to fishbone ingestion was successfully treated through combined TEVAR and VATS (13). It seemed that combining TEVAR with VATS treatment in a less invasive way was effective and safer compared with emergent open surgery.

As our patient was admitted with sentinel hemorrhage, sepsis, and mediastinitis, we performed a salvaged TEVAR immediately as the first step to avoid fatal hemorrhage. For open thoracic repair, surgery would not have been appropriate based on the condition of our patient. Complete mediastinal drainage could prevent the thoracic aorta stenting from secondary infection or reduce the risk of aortic rupture, so we gave the patient a lateral cervical incision and transcervical mediastinal debridement and drainage under general anesthesia, combined with thoracoscopic debridement and drainage with VATS later.

Since the perforation of the esophagus is not repaired and the treatment of esophageal mediastinal leakage requires long-term fasting, nasogastric feeding, or gastrostomy reflux should be avoided to aggravate the mediastinal infection. Hence, jejunostomy was performed. Individualized nutrition programs were also formulated for the patient. The patient was given antibiotics after transferring to our hospital and this treatment lasted for 5 weeks. After being discharged, he was in a stable condition and did not continue antibiotic treatment. In this case, systematic treatment with nutritional support and effective antibiotics was also key to saving the patient’s life.

From our experience and based on the reports of other researchers, it is not difficult to ascertain that TEVAR combined with VATS in a less-invasive way seems to be effective and much safer compared to previous open surgery. However, more data are needed to confirm its effectiveness and safety in the future. For this patient, we used a staged approach with salvage TEVAR followed by VATS, while some researchers combined TEVAR and VATS in one stage. We had also considered the one-stage hybrid treatment of TEVAR and VATS for our patients, but we chose to first perform salvage TEVAR and then the second stage of VATS, with a lateral cervical incision for fully mediastinal debridement and drainage, leaving some time for antibiotic treatment and improvement of his general condition, since the patient was suffering from a severe mediastinitis and fever when he was admitted to our hospital. For severely infected AEFs caused by EFB, we believe this salvaged staged approach is safer and has a higher success rate. However, no matter which strategy is used, the most important principles are the same: salvaged TEVAR and thorough mediastinal debridement and drainage are preferable.

## Conclusion

Patients suffering from EFB should be carefully assessed with CT to determine the risk of AEF, especially when the EFB is located in the thoracic esophagus, before and after EFB removal. Additionally, the staged strategy of combing salvaged TEVAR with subsequent adequate mediastinal debridement and drainage in a less invasive approach with systematic treatment may be an alternative and provide safer care for patients with AEF infections caused by EFB.

## Data Availability

The original contributions presented in the study are included in the article/supplementary material, further inquiries can be directed to the corresponding author.
